# Bullying victimization and depressive symptoms among left-behind children: a moderated mediation model involving Internet Addiction

**DOI:** 10.3389/fpsyt.2026.1826121

**Published:** 2026-06-12

**Authors:** Qi Li, Gexuan Wang, Jiannan Zheng, Ruifeng Liu

**Affiliations:** 1 School of Physical Education, Central China Normal University, Wuhan, China; 2 College of Professional Tennis, Wuhan City Polytechnic, Wuhan, China; 3 School of Physical Education, Hubei University, Wuhan, China

**Keywords:** bullying victimization, depressive symptoms, Internet Addiction, left-behind children, mental health, moderated mediation analysis

## Abstract

**Objective:**

This study aimed to examine the mediating role of Internet Addiction in the association between bullying victimization and Depressive Symptoms among left-behind children, as well as the moderating role of physical activity in this relationship.

**Methods:**

A cross-sectional design was adopted. A total of 21,689 left-behind children were recruited using stratified random sampling. A moderated mediation model was constructed within the generalized linear model framework. Conditional indirect effects and the probability scale were further used to analyze the relationships among bullying victimization, Internet Addiction, physical activity, and Depressive Symptoms in left-behind children.

**Results:**

After controlling for confounding factors, bullying victimization significantly predicted Depressive Symptoms among left-behind children (B = 1.179, p < 0.001). Internet Addiction partially mediated the association between bullying victimization and Depressive Symptoms. Specifically, bullying victimization significantly predicted Internet Addiction among left-behind children (B = 1.041, p < 0.001), and Internet Addiction significantly predicted Depressive Symptoms (B = 1.218, p < 0.001). Moreover, physical activity significantly moderated the association between bullying victimization and Internet Addiction (p < 0.05).

**Conclusion:**

Among left-behind children, bullying victimization may increase the risk of Depressive Symptoms partly by triggering Internet Addiction, while physical activity may buffer the association between bullying victimization and Internet Addiction.

## Introduction

Depressive Symptoms, as a subclinical stage of depression, are commonly used for early monitoring and screening of depression because the corresponding assessment tools are easy to administer and cost-effective (1). These symptoms are considered part of the severity continuum of unipolar depressive disorder, and different stages of this continuum are associated with impairments in psychosocial functioning (2, 3). The resulting adverse outcomes are similar to those associated with depression, including self-harm or suicidal behavior (4), substance abuse (5, 6), social problems (7), and reduced educational attainment (8). Based on findings from multiple meta-analyses, the prevalence of Depressive Symptoms among Chinese children and adolescents is approximately 20%–26.2% (1, 9, 10). Another meta-analysis reported that, as of 2024, the global prevalence of Depressive Symptoms among adolescents was approximately 21.3% (11). These findings echo the concern of the World Health Organization that Depressive Symptoms are among the most common mental health problems in adolescents (12). However, the above data mainly focus on the general adolescent population. In China, there is a special group known as left-behind children, which is a sociological and demographic label rather than an age-stage category. The emergence of left-behind children is closely related to economic and resource disparities between urban and rural areas, as well as across regions in China. Many parents migrate to economically developed areas for work in pursuit of higher income, while their children are usually cared for by one parent or other relatives (13). Owing to the unique living circumstances of this group, left-behind children are more vulnerable to mental health problems when facing stress or challenges (14, 15). Previous research has further shown that the prevalence of Depressive Symptoms among left-behind children is 1.7 times that among non-left-behind children (16).

The onset and progression of Depressive Symptoms are influenced by multilevel factors (17, 18). In China’s K–12 education system, students must pass entrance examinations, such as the senior high school entrance examination and the national college entrance examination, to progress to the next stage of education. Although this system formally provides equal educational opportunities for all students, access to high-quality educational resources remains limited. As a result, many students must stand out in intense competition and therefore experience substantial academic pressure (19–21). Excessive academic burden reduces students’ time for free activities and social interaction (22), making their social activities largely confined to the school educational environment (4). Evidence suggests that healthy social relationships can provide adolescents with psychological support and help them cope more effectively with negative emotions (23, 24). In contrast, adolescents who experience maladaptive interpersonal interactions or bullying victimization are more likely to develop Depressive Symptoms (25–27), and interpersonal conflicts are more prevalent among left-behind children (14, 28).

Bullying is a public health issue of global concern (29). Specifically, bullying refers to repeated and intentional harmful behaviors perpetrated by an individual or group against a person who is relatively disadvantaged in terms of physical or psychological power. It generally includes two dimensions: victimization and perpetration. Previous studies have shown that perpetrators, victims, and bully-victims may all experience adverse mental health outcomes (30). Compared with the other two roles, however, bullying-victimized adolescents tend to experience more severe and persistent mental health impairments (31, 32).

A meta-analysis showed that approximately one-quarter of students worldwide have experienced bullying victimization (33). Extensive evidence indicates that bullying victimization is associated with various adverse health outcomes, such as depression (34), Internet Addiction (35), and self-harm behavior (36).

Internet Addiction refers to an individual’s excessive use of the Internet (37). It is characterized by strong psychological dependence and difficulty in self-regulating Internet-related behaviors (38), and may affect physical and mental health through biological, sociological, and psychological mechanisms (39). According to the Quality of (Real) Life Hypothesis, individuals who are frequently exposed to stressful or frustrating environments are more likely to use the Internet as a means of escaping stress and obtaining psychological compensation (40). This is consistent with the self-medication hypothesis, which suggests that addictive behaviors may represent a maladaptive response to negative emotions (41). In line with these theories, left-behind children often lack parental emotional support and care for much of their development, which may lead to distorted worldviews, life views, and values, as well as abnormal psychological development. Consequently, they may be more likely to use the Internet as a coping strategy for stress (42). Previous studies have also supported a positive association between bullying victimization and Internet Addiction (43–45). Therefore, Internet Addiction may be both a risk factor for Depressive Symptoms and a consequence of bullying victimization.

Unfortunately, the mental health status of these relatively disadvantaged groups in Chinese society has not received sufficient attention. Existing studies on the mental health of left-behind children have mostly focused on descriptive comparisons or the identification of risk factors. Although previous research has shown that mental health problems are more severe among left-behind children than among non-left-behind children (46, 47), systematic empirical analyses remain limited regarding the structural relationships among factors associated with Depressive Symptoms within the left-behind child population, particularly the potential mediating association and patterns among bullying victimization, Internet Addiction, and Depressive Symptoms.

Beyond the above risk pathway, it is also important to identify protective factors that may weaken or buffer these negative effects. The conceptual model of “sport for health” suggests that participation in sports can bring multiple psychological and social health benefits to children and adolescents. A cross-sectional study reported that sports participation can buffer the effects of being bullied and may serve as an effective strategy for increasing adolescents’ physical activity, positive peer interaction, and positive emotions (48). One possible explanation is that physical activity regulates the HPA axis and stress responses, enhances emotion regulation, and provides social and behavioral protection (49). Moreover, sufficient physical activity may reduce the predictive effect of bullying victimization on Internet Addiction among adolescents (50). The possible mechanisms may involve cognitive and emotional regulation, social belonging, and behavioral substitution. Specifically, physical activity may enhance self-control and emotion regulation, thereby reducing excessive Internet use driven by negative emotions or impulsivity (51, 52). In addition, physical activity provides real-world social connection and emotional satisfaction, meets adolescents’ need for belonging, and promotes prosocial behaviors that can substitute for the gratification obtained from virtual social interaction, thereby further reducing the risk of Internet Addiction (53, 54). This mechanism may be particularly important for left-behind adolescents who lack sufficient emotional support. Insomnia caused by Internet Addiction is associated with impaired neuroplasticity, which may further aggravate Depressive Symptoms (55). Sufficient physical activity is thought to promote AMPA receptor signaling and improve plasticity by acting on important targets such as BDNF. This mechanism shows some similarity to the rapid plasticity remodeling induced by ketamine (56, 57).

**Figure 1:**
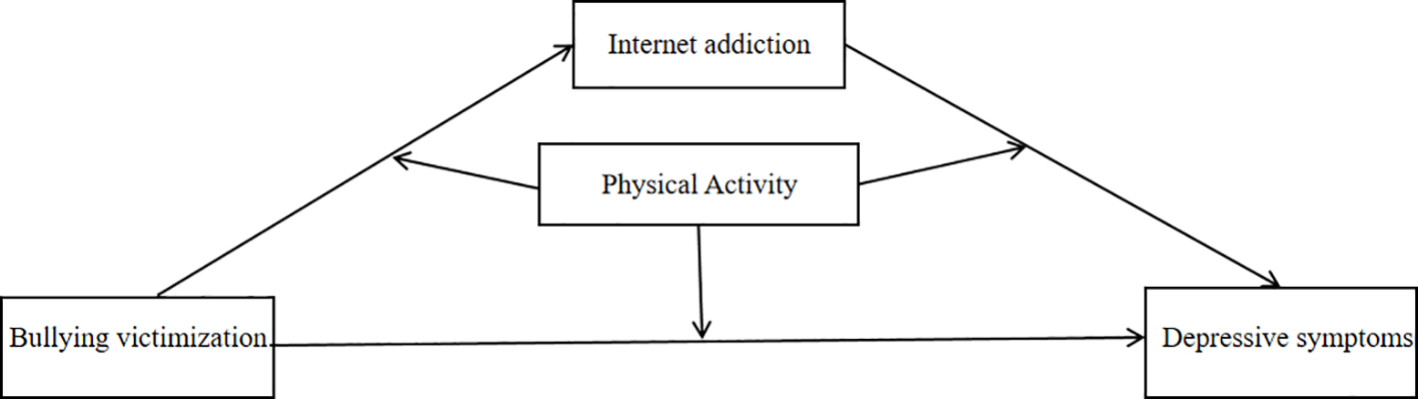
Hypothesized moderated mediation model.

Therefore, based on the above evidence and the gaps in existing research, we proposed the following hypotheses and constructed a moderated mediation model, as shown in **Figure 1**: (1) bullying victimization would be positively associated with Depressive Symptoms among left-behind adolescents; (2) Internet Addiction would mediate the association between bullying victimization and Depressive Symptoms; and (3) participation in physical activity would moderate the relationships among bullying victimization, Internet Addiction, and Depressive Symptoms. Specifically, physical activity may moderate the effect of bullying victimization on Internet Addiction, the effect of Internet Addiction on Depressive Symptoms, and the direct effect of bullying victimization on Depressive Symptoms.

## Methods

### Participants

This study used cross-sectional data from the 2022–2023 Hubei Province Surveillance Program on Common Diseases and Health Influencing Factors among Students. Before being provided to the research team, all data were thoroughly anonymized by the surveillance management authority and contained no personally identifiable information. The study protocol was approved by the Ethics Committee of the Hubei Provincial Center for Disease Control and Prevention (Approval No. 2024-016-01) and was conducted in accordance with the ethical principles of the Declaration of Helsinki. A stratified cluster random sampling method was used, covering 13 prefecture-level cities/prefectures, as well as four directly administered county-level cities and forest districts across the province. In each prefecture-level city/prefecture, seven schools were selected, including four urban schools—two junior high schools and two senior high schools—and three county-level schools—two junior high schools and one senior high school. Within each school, grades were used as strata and classes as sampling units, and intact classes were randomly selected from each grade to participate in the survey. In principle, at least three classes were selected from each school, with an expected sample size of approximately 240 students per school. If the number of students in an individual school was insufficient, additional participants were recruited from schools in the same area, educational stage, and school type to ensure that the sample size at each stratum met the study design requirements. Based on participants’ self-reported questionnaire information, such as responses to the question “Which family members have lived with you in the past six months?”, participants were classified as left-behind children if one or both of their parents had migrated for work and had been away from home for at least six consecutive months.

A total of 29,340 participants were initially included in this study. After data screening, 7,349 participants who reported that they had never used the Internet were excluded, along with 204 participants with missing or incomplete information on Depressive Symptoms or Internet Addiction, 84 participants with missing physical activity data, and 14 participants with missing demographic covariates. Ultimately, 21,689 participants were included in the final analysis.

#### Inclusion criteria

1. Students who participated in the Surveillance Program on Common Diseases and Health Influencing Factors among Students;2.Children who met the criteria for being classified as left-behind children, namely those whose father, mother, or both parents had been away from home for at least six consecutive months and who were cared for by one parent, grandparents, or other guardians;3.Participants who completed the questionnaire required for this study and had complete information on the main study variables and covariates.

#### Exclusion criteria

1. Participants who reported that they had never used the Internet and therefore could not be assessed for Internet Addiction symptoms;2.Participants with missing or incomplete information on major study variables, including bullying victimization, Depressive Symptoms, Internet Addiction, physical activity, and demographic covariates;3.Participants whose questionnaire responses showed obvious logical inconsistencies or insufficient overall completeness.

### Measurement tools

#### Bullying victimization

Bullying victimization was assessed using six items from the revised Olweus Bully/Victim Questionnaire (58). The questionnaire covers verbal bullying, relational bullying, and physical bullying occurring in the past 30 days. Each item was rated on a three-point scale (0 = never, 1 = sometimes, 2 = often). Participants who selected “sometimes” or “often” for any item were classified as victims of bullying. This instrument has been validated and widely used in studies of Chinese adolescents, showing good reliability and validity (59–61).

#### Internet addiction

Participants’ Internet Addiction symptoms were assessed using a scale revised based on relevant criteria from the Diagnostic and Statistical Manual of Mental Disorders, Fifth Edition (DSM-5) (62), with reference to the Internet Addiction scale developed by Tao Ran (63). Each item was scored dichotomously (0 = no, 1 = yes). For example, one item was “I often use the Internet, and even when I am not online, Internet-related thoughts keep coming to my mind.” The total score for Internet Addiction ranged from 0 to 9, and participants with a score ≥4 were classified as having Internet Addiction (64). This scale is the designated instrument used in the National Surveillance Program on Common Diseases and Health Influencing Factors among Students in China. It has been widely applied in surveillance surveys across multiple provinces and has demonstrated good reliability and validity (65–67). In the present sample, the internal consistency of the scale, as assessed by the Kuder–Richardson Formula 20 (KR-20), was 0.783, indicating acceptable reliability.

#### Depressive symptoms

Depressive Symptoms were assessed using the Center for Epidemiologic Studies Depression Scale (CES-D) (68). The scale consists of 20 items, including statements such as “I was bothered by things that usually do not bother me” and “I did not feel like eating; my appetite was poor.” Each item was rated on a four-point scale according to symptom frequency: 0 = less than 1 day, 1 = 1–2 days, 2 = 3–4 days, and 3 = 5–7 days. Items 4, 8, 12, and 16 are positively worded and were reverse-scored. The total score was calculated by summing all item scores, with higher scores indicating more severe Depressive Symptoms. Participants with a CES-D total score ≥16 were classified as having Depressive Symptoms (69). In this study, the scale showed good internal consistency (Cronbach’s α = 0.895). In addition, its structural stability and psychometric properties have been widely validated in Chinese adolescent samples (70, 71).

#### Physical activity level

Participants’ physical activity level was assessed according to the WHO 2020 Guidelines on Physical Activity and Sedentary Behaviour for adolescents. Based on the self-reported physical activity questionnaire, accumulating fewer than 3 days of moderate-to-vigorous physical activity per week was defined as insufficient physical activity, indicating a lack of exercise behavior (72). Physical activity was coded as a binary variable: 0 indicated sufficient physical activity, and 1 indicated insufficient physical activity.

#### Covariates

Participants’ Age, Gender, Grade, Area, and other demographic variables were included as covariates in this study. These data were obtained from participants’ self-reported questionnaires, and some variables were recoded into categorical variables for analysis.

#### Statistical analyses

Descriptive statistics were used to summarize the basic characteristics of the participants. Continuous variables were presented as means ± standard deviations (SD), while categorical variables were presented as frequencies and percentages. Between-group differences were compared using *t* tests for continuous variables and chi-square (χ²) tests for categorical variables.

To preliminarily examine the bivariate relationships among bullying victimization, Internet Addiction, Depressive Symptoms, and physical activity level, partial correlation analyses were conducted, controlling for covariates including BMI, Age, Gender, Area, and Grade.

Finally, to test the study hypotheses, a moderated mediation model was constructed using Mplus version 8.3 (73). The model was used to examine the association between bullying victimization and Depressive Symptoms among left-behind children, the mediating role of Internet Addiction, and the moderating role of physical activity. Given that the dependent variables in the model were binary, binary logistic regression models within the generalized linear model framework were applied (74), with the logit function specified as the link function and parameters estimated using maximum likelihood estimation. The logit link function transforms the probability of event occurrence into the log odds, enabling the model to characterize the nonlinear relationship between independent variables and event probabilities (75). Path coefficients in the model represent unstandardized regression coefficients on the logit scale. Indirect effects and conditional indirect effects were tested using 2,000 bootstrap resamples, and bias-corrected 95% confidence intervals were reported. Furthermore, after significant moderating effects were detected, the magnitude and direction of the moderating effects were evaluated using the difference in conditional indirect effects (DIFFIND) and the difference in risk differences (DID) on the probability scale. Given that coefficients on the logit scale do not correspond linearly to effects on the probability scale, the related results were interpreted with caution. BMI, Age, Gender, Area, and Grade were included as covariates in the model. All statistical tests were two-sided, and *p* < 0.05 was considered statistically significant.

## Results

### Description of participants’ characteristics

A total of 21,689 participants were included in this study, with a mean age of 14.387 ± 1.705 years. As shown in **Table 1**, 8,665 participants were boys (43.79%) and 12,196 were girls (56.21%). Most participants were general secondary school students (n = 20,366, 93.86%), while a smaller proportion were Vocational High School students (n = 1,332, 6.14%). Among all participants, 5,969 (27.51%) reported bullying victimization in the past 30 days. The proportions of these participants in the sufficient and insufficient physical activity groups were 39.22% and 60.78%, respectively. A total of 2,684 participants (12.37%) were classified as having Internet Addiction, with 52.42% in the sufficient physical activity group and 47.58% in the insufficient physical activity group. In addition, 8,612 participants (39.69%) had Depressive Symptoms, with 43.89% and 56.11% in the sufficient and insufficient physical activity groups, respectively. The mean Internet Addiction total score was 1.264 ± 1.844 in the overall sample, 1.066 ± 1.678 in the sufficient physical activity group, and 1.561 ± 2.032 in the insufficient physical activity group. The mean CES-D total score was 14.460 ± 8.381 overall, 14.337 ± 8.149 in the sufficient physical activity group, and 14.644 ± 8.714 in the insufficient physical activity group.

**Table 1:** Baseline characteristics of participants stratified by sufficiency of physical activity (N=21698).

Variable	Total (N = 21698)	Sufficient physical activity (n =8665) M (SD)/n (%)	Physical inactivity (n = 13032) M (SD)/n (%)	χ2/t test
Age	14.387 ± 1.705	14.666 ± 1.767	14.202 ± 1.636	19.493***
BMI	21.175 ± 3.882	21.289 ± 3.931	21.099 ± 3.848	3.523***
Gender				0.229
Female	12196(56.21%)	4853(39.79%)	7343(60.21%)	
Male	9501(43.79%)	3812(40.12%)	5689(59.88%)	
Grade				609.243***
Junior High School	12143(55.96%)	3968(32.68%)	8175(67.32%)	
Senior High School	8223(37.90%)	4010(48.77%)	4213(51.23%)	
Vocational High School	1332(6.14%)	687(51.58%)	645(48.42%)	
Area				47.343***
rural	14025(64.64%)	5363(38.24%)	8662(61.76%)	
Urban	7673(35.36%)	3302(43.03%)	4371(56.97%)	
Bullying victimization				1.715
YES	5969(27.51%)	2341(39.22%)	3628(60.78%)	
NO	15729(72.49%)	6324(40.21%)	9405(59.79%)	
Internet Addiction				198.513***
YES	2684(12.37%)	1407(52.42%)	1277(47.58%)	
NO	19014(87.63%)	7258(38.17%)	11756(61.83%)	
Depressive Symptoms				92.972***
YES	8612(39.69%)	3780(43.89%)	4832(56.11%)	
NO	13086(60.31%)	4885(37.33%)	8201(62.67%)	
Internet Addiction Total Score	1.264 ± 1.844	1.066 ± 1.678	1.561 ± 2.032	18.831***
Depressive Symptoms Total Score	14.460 ± 8.381	14.337 ± 8.149	14.644 ± 8.714	2.613**

M, mean; SD, Standard Deviation. ***p<0.001, **p<0.01, *p<0.05.

### Correlation analysis of key variables

As shown in **Table 2**, after adjusting for covariates including Age, Grade, and Gender, significant associations were observed among the four key variables: bullying victimization, Internet Addiction, Depressive Symptoms, and physical activity.

**Table 2:** Covariate-adjusted pairwise associations among key variables.

Variable	1	2	3	4
Bullying victimization	-			
Internet Addiction	0.122***	-		
Depressive Symptoms	0.228***	0.183***	-	
Physical Activity	0.019**	-0.082***	-0.031***	-

Bullying victimization was coded as 0 = no and 1 = yes; physical activity was coded as 0 = insufficient and 1 = sufficient; depressive symptoms were coded as 0 = no and 1 = yes; and Internet addiction was coded as 0 = no and 1 = yes. Values are partial correlation coefficients controlling for BMI, age, gender, area, and grade. All estimates are rounded to three decimal places. ***p<0.001, **p<0.01, *p<0.05.

### Mediation analyses

**Figure 2:**
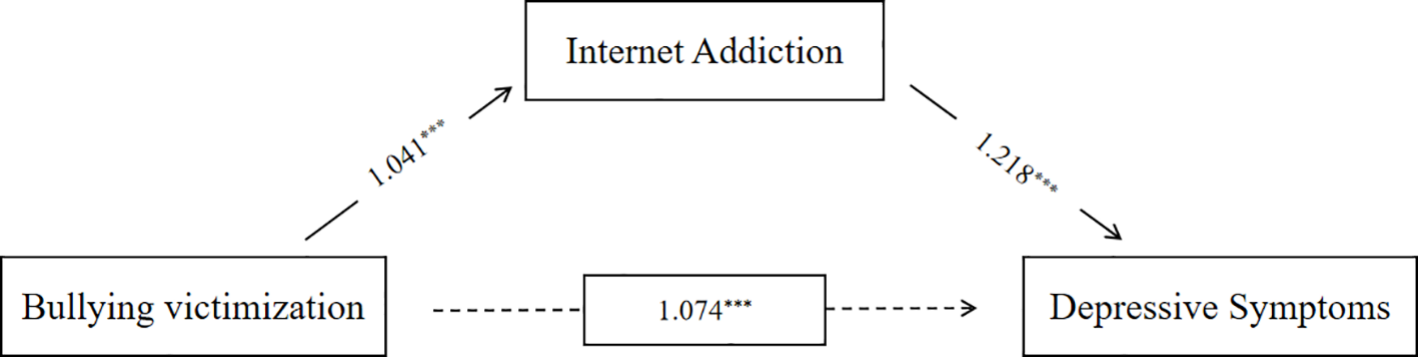
Simple mediation model. Values shown on the paths are unstandardized logit coefficients (B). For paths with binary dependent variables, coefficients were estimated using logistic regression models. All models controlled for BMI, age, gender, area, and grade. ***p<0.001, **p<0.01, *p<0.05.

As shown in **Table 3**, bullying victimization was significantly positively associated with Depressive Symptoms among left-behind children (B = 1.179, SE = 0.032, p < 0.001). After including the mediator, Internet Addiction, the association between bullying victimization and Depressive Symptoms remained statistically significant (B = 1.074, SE = 0.033, p < 0.001).Meanwhile, bullying victimization was significantly positively associated with Internet Addiction (B = 1.041, SE = 0.041, p < 0.001), and Internet Addiction was also significantly positively associated with Depressive Symptoms (B = 1.218, SE = 0.046, p < 0.001). Detailed path effects are presented in **Figure 2** and **Table 4**. Therefore, Hypotheses 1 and 2 were supported.

**Table 3:** Construction and parameter estimates of simple mediation model.

Outcome variable	Predictor variable	McFaddenR²	LRχ²	B (SE)	OR [95% CI]	P value
Depressive Symptoms		0.060	1759.593***			
	Age			0.179(0.014)	1.196[1.164,1.230]	<0.001
	BMI			0.000(0.004)	1.000[0.993,1.008]	0.912
	Grade			0.049(0.040)	1.050[0.970,1.134]	0.215
	Gender			-0.118(0.030)	0.889[0.835,0.938]	<0.001
	Area			-0.052(0.032)	0.949[0.890,1.013]	0.106
	Bullying victimization(X)			1.179(0.032)	3.251[3.050,3.452]	<0.001
Internet Addiction		0.043	697.257***			
	Age			0.124(0.019)	1.132[1.089,1.175]	<0.001
	BMI			0.003(0.006)	1.003[0.992,1.014]	0.596
	Grade			0.021(0.057)	1.021[0.915,1.143]	0.715
	Gender			0.162(0.045)	1.176[1.079,1.284]	<0.001
	Area			-0.014(0.048)	0.986[0.899,1.083]	0.768
	Bullying victimization(X)			1.041(0.041)	2.832[2.601,3.062]	<0.001
Depressive Symptoms		0.086	2501.414***			
	Age			0.168(0.014)	1.183[1.154,1.218]	<0.001
	BMI			0.000(0.004)	1.000[0.992,1.008]	0.990
	Grade			0.049(0.040)	1.050[0.970,1.133]	0.227
	Gender			-0.144(0.030)	0.866[0.814,0.917]	<0.001
	Area			-0.053(0.033)	0.948[0.889,1.014]	0.113
	Bullying victimization(X)			1.074(0.033)	2.927[2.746,3.114]	<0.001
	Internet Addiction(M)			1.218(0.046)	3.380[3.086,3.691]	<0.001

***p<0.001, **p<0.01, *p<0.05.

**Table 4:** Effect decomposition and bootstrap 95% confidence interval (95% CI).

Effect	Estimate(SE)	Bootstrap 95% CI	P value
Indirect effect, a×b	1.268(0.069)	[1.126,1.399]	<0.001
Direct effect, c′	1.074(0.033)	[1.010,1.136]	

Estimates are reported on the logit scale. The indirect effect was calculated using the product-of-coefficients approach. The direct effect c′ represents the effect of bullying victimization on depressive symptoms after adjusting for internet addiction. Bootstrap resampling was used to estimate the 95% confidence intervals. ***p<0.001, **p<0.01, *p<0.05.

### Moderated mediation analyses

As shown in **Table 5**, after including physical activity as a moderating variable, bullying victimization remained a significant positive predictor of Internet Addiction risk among left-behind children (B = 0.959, SE = 0.059, p < 0.001), while physical activity significantly negatively predicted Internet Addiction risk (B = −0.642, SE = 0.057, p < 0.001).In addition, the interaction term between bullying victimization and physical activity was a significant predictor of Internet Addiction risk (B = 0.179, SE = 0.084, p < 0.05), indicating that physical activity significantly moderated the association between bullying victimization and Internet Addiction. However, in the model with Depressive Symptoms as the outcome, the interaction term between bullying victimization and physical activity was not statistically significant (B = 0.078, SE = 0.068, p > 0.05), and the interaction between Internet Addiction and physical activity was also non-significant (B = 0.131, SE = 0.092, p > 0.05). These findings suggest that physical activity did not significantly moderate either the direct effect of bullying victimization on Depressive Symptoms or the effect of Internet Addiction on Depressive Symptoms. Further analysis of conditional indirect effects (**Table 6**) showed that the difference in the conditional indirect effect linking bullying victimization to Depressive Symptoms via Internet Addiction between the insufficient and sufficient physical activity groups reached statistical significance (p < 0.05).Given that the above conditional indirect effects were estimated based on the product of path coefficients on the logit scale, this study further interpreted the moderating role of physical activity in the association between bullying victimization and Internet Addiction from the perspectives of predicted probabilities and risk differences, in order to reflect differences in this moderated pathway at the practical risk level. As shown in **Table 7**, for Internet Addiction, the difference in risk differences between the two groups was statistically significant (DID = −0.026, 95% CI [−0.051, −0.003], p < 0.05). This finding further indicates that the association between bullying victimization and the risk of Internet Addiction differs significantly at the probability scale across levels of physical activity. In terms of direction, sufficient physical activity may exert a buffering effect.

**Table 5:** Results of the moderating effect of physical activity on the mediation model.

Predictor variable	Outcome variable: internet addiction			Outcome variable: depressive symptoms		
	B (SE)	OR [95% CI]	P value	B (SE)	OR [95% CI]	P value
Age	0.119(0.020)	1.126[1.083,1.168]	<0.001	0.167(0.014)	1.182[1.153,1.217]	<0.001
BMI	0.004(0.005)	1.004[0.993,1.015]	0.427	0.000(0.004)	1.000[0.993,1.008]	0.921
Grade	–0.049(0.058)	0.953[0.850,1.068]	0.406	0.031(0.041)	1.031[0.950,1.114]	0.452
Gender	0.188(0.045)	1.207[1.108,1.323]	<0.001	–0.138(0.030)	0.871[0.819,0.923]	<0.001
Area	–0.010(0.048)	0.990[0.902,1.088]	0.832	–0.051(0.033)	0.950[0.890,1.016]	0.124
Bullying victimization (X)	0.959(0.059)	2.609[2.323,2.912]	<0.001	1.029(0.052)	2.797[2.513,3.097]	<0.001
Physical Activity (W)	–0.642(0.057)	0.526[0.467,0.591]	<0.001	–0.190(0.037)	0.827[0.768,0.888]	<0.001
Bullying victimization (X)*Physical Activity (W)	**0.179(0.084)**	**1.196[1.018,1.416]**	**<0.05**	0.078(0.068)	1.081[0.940,1.231]	0.251
Internet Addiction (M)				1.134(0.065)	3.107[2.705,3.508]	<0.001
Internet Addiction (M)*Physical Activity (W)				0.131(0.092)	1.140[0.964,1.380]	0.155
LR χ²	875.412***			2529.832***		
McFadden R²	0.054			0.087		

***p<0.001, **p<0.01, *p<0.05.

Bold values indicate statistically significant results.

**Table 6:** Conditional indirect effects of physical activity moderating related pathways and their differences.

Moderator (W)	Indirect effect (IND) [95% CI]	Difference (DIFFIND) [95% CI]	P value
Insufficient physical activity (W = 0)	1.087[0.910,1.274]		
Sufficient physical activity (W = 1)	1.439[1.252,1.667]	0.352[0.079,0.648]	**<0.05**

IND = a × b, representing the indirect effect on the log-odds scale. DIFFIND = IND(W = 1) − IND(W = 0), representing the index of moderated mediation. 95% CIs were bias-corrected bootstrap confidence intervals. All covariates were held constant at their sample means.*p<0.05.

Bold values indicate statistically significant results.

**Table 7:** Predicted probabilities and risk differences of bullying victimization for Internet addiction across different physical activity levels.

Outcome variable	Condition/Index	Bullying victimization (X1 = 0), P [95% CI]	Bullying victimization (X1 = 1), P [95% CI]	Risk difference (RD) [95% CI]	Difference in risk differences (DID) [95% CI]	P value
Internet addiction (M)						
	W = 0 (insufficient)	0.119[0.112,0.127]	0.260[0.244,0.278]	0.141[0.123,0.159]		
	W = 1 (sufficient)	0.066[0.061,0.071]	0.181[0.169,0.194]	0.115[0.101,0.128]	**–0.026[–0.051,–0.003]**	**<0.05**
Depressive Symptoms(Y)						
	W = 0 (insufficient)	0.348[0.336,0.360]	0.625[0.605,0.645]	0.277[0.253,0.300]		
	W = 1 (sufficient)	0.296[0.286,0.305]	0.584[0.568,0.601]	0.288[0.269,0.307]	0.011[–0.019,0.040]	0.458

P denotes the model-predicted probability of the outcome, estimated at the specified levels of bullying victimization and physical activity, with all other covariates held at their sample means. RD = P(X1 = 1) − P(X1 = 0), representing the absolute risk difference between bullying and no bullying. DID = RD(W = 1) − RD(W = 0), representing the interaction effect on the probability scale. The 95% CIs were obtained using bias-corrected bootstrap resampling.

Bold values indicate statistically significant results.

## Discussion

Our findings indicate that Internet Addiction partially mediated the association between bullying victimization and Depressive Symptoms among left-behind children. This suggests that bullying victimization not only directly increases the risk of Depressive Symptoms in this population, but may also indirectly exacerbate Depressive Symptoms by increasing the level of Internet Addiction. In addition, physical activity showed a significant moderating effect on the pathway between bullying victimization and Internet Addiction. Specifically, sufficient physical activity may exert a buffering effect on the association between bullying victimization and Internet Addiction.

In the present study, bullying victimization significantly predicted the risk of Depressive Symptoms among left-behind children and was also significantly associated with Internet Addiction. Although these pathways have been widely supported in general adolescent populations (4, 76, 77), they may manifest with greater vulnerability among left-behind children. According to family systems theory (78), interactions and supportive behaviors within the family system play a critical role in adolescents’ social adaptation and psychological development. However, left-behind children often experience prolonged absence of one or both parents, which may constrain the development of emotional buffering and regulation capacities, thereby amplifying the negative impact of bullying victimization on mental health. From the perspective of the compensatory Internet use model (79), individuals who experience adverse life events, such as bullying victimization, may increase Internet use to compensate for unmet emotional and social needs. In this context, Internet use may serve as a temporary coping strategy to alleviate distress or vulnerability (80). However, such “compensatory use” is often associated with a higher risk of addiction, particularly among individuals with pre-existing psychosocial difficulties. This process may contribute to a reinforcing cycle of “negative emotions – excessive Internet use – exacerbation of negative emotions” (79). In this study, Internet Addiction partially mediated the association between bullying victimization and Depressive Symptoms. For left-behind children, the lack of parental companionship, limited development of regulatory capacities, and exposure to multiple real-life stressors may increase reliance on the Internet as a primary compensatory outlet, thereby elevating the risk of Internet Addiction. Taken together, it is therefore unsurprising that Internet Addiction exhibits a significant mediating role in the association between bullying victimization and Depressive Symptoms.

Our results further indicated that physical activity significantly moderated the pathway between bullying victimization and Internet Addiction, whereas the moderating effects on the pathways between Internet Addiction and Depressive Symptoms, as well as between bullying victimization and Depressive Symptoms, were not statistically significant. This pattern may reflect that, in the specific context of left-behind children, the buffering and protective effects of physical activity on negative emotions are limited. As noted earlier, physical activity among left-behind children predominantly occurs within the school environment, which is also a high-risk setting for bullying victimization. Therefore, physical activity may not necessarily provide sufficient social interaction or emotional support in this context (81). For left-behind children experiencing bullying victimization, socially interactive physical activity settings may instead be accompanied by exclusion, threat, or social comparison (82, 83), thereby limiting the buffering effects typically reported in the literature. Moreover, physical activity in school settings is often structured and, to some extent, compulsory, and may not yield the same positive emotional experiences or high-quality social support as activities that are voluntarily chosen and conducted with family members or friends during weekends or holidays (84). Such support from family or stable peers is precisely what left-behind children tend to lack over extended periods, which may further constrain the emotional buffering function of physical activity in bullying contexts. Although physical activity may theoretically counteract Internet Addiction–related sleep disturbances and impaired neuroplasticity by promoting neurotrophic factors such as BDNF (85, 86), the intensity and quality of these structured group activities may be insufficient to reach the threshold required to effectively activate such neuroprotective mechanisms in left-behind children (87). In addition, potential negative social experiences within these activity settings may induce elevated cortisol levels, which can suppress BDNF expression (88), thereby offsetting the potential physiological benefits of physical activity. Consequently, physical activity may fail to significantly attenuate the effect of Internet Addiction on Depressive Symptoms. On the other hand, for left-behind children experiencing bullying victimization, limited access to supportive resources and restricted channels for emotional expression and release may lead them to rely more heavily on Internet use as a means of escaping real-life stress and obtaining immediate relief or compensatory satisfaction (80, 89, 90). Although this form of physical activity may not be sufficient to repair bullying-related damage to self-esteem, interpersonal insecurity, or Depressive Symptoms, it may still temporarily divert attention away from bullying victimization, alleviate tension and psychological distress, and occupy time that might otherwise be spent on excessive Internet use. In this way, physical activity may partially buffer the effect of bullying victimization on Internet Addiction. This may also explain why, in the present study, physical activity moderated the pathway between bullying victimization and Internet Addiction, but did not significantly moderate the pathways between bullying victimization and Depressive Symptoms or between Internet Addiction and Depressive Symptoms.

The moderated mediation model constructed in this study provides new insights into understanding the association between bullying victimization and Depressive Symptoms among left-behind children, as well as potential coping strategies. By identifying the mediating role of Internet Addiction and the moderating role of physical activity, this study highlights the need for schools and communities not only to strengthen bullying prevention efforts but also to promote healthy Internet use among left-behind children, thereby reducing the reciprocal reinforcement between negative emotions and excessive Internet use. In addition, targeted attention should be given to left-behind children with limited social support, weak peer relationships, or a higher risk of bullying victimization. Encouraging participation in appropriate and supportive physical activity may help expand channels for emotional expression and stress relief, thereby reducing reliance on the Internet as a means of escaping real-life stress. Overall, the findings underscore the unique challenges faced by left-behind children exposed to bullying victimization and provide an important basis for developing tailored mental health protection strategies and support systems for this population.

## Conclusion

This study further elucidated the association between bullying victimization and Depressive Symptoms among left-behind children by incorporating the mediating role of Internet Addiction and the moderating role of physical activity. The findings indicate that Internet Addiction partially mediates the relationship between bullying victimization and Depressive Symptoms, while physical activity significantly moderates the pathway between bullying victimization and Internet Addiction. These results suggest that schools and communities should promptly identify whether left-behind children have experienced bullying victimization and provide timely support to alleviate negative emotions, thereby safeguarding their mental health and overall well-being.

## Strengths

This study has several notable strengths. First, to our knowledge, it is the first study to construct a moderated mediation model in a large sample of left-behind children in China to examine the association between bullying victimization and Depressive Symptoms. In addition, a stratified random sampling approach was employed across the entire province, with the sample covering junior high schools, general senior high schools, and Vocational High Schools. This enhances the applicability of the findings across different educational settings. By focusing on left-behind children, a high-risk population, the study contributes to a deeper understanding of heterogeneity in the development of mental health risks among adolescents and provides practical implications for targeted interventions. Second, this study employed well-validated measurement instruments. These included an Internet Addiction assessment tool revised with reference to criteria from the Diagnostic and Statistical Manual of Mental Disorders, Fifth Edition (DSM-5), as well as widely validated measures of mental health and behavior, such as the Center for Epidemiologic Studies Depression Scale (CES-D) and the WHO physical activity guidelines. The use of these instruments enhances the reliability of variable measurement and the comparability of findings. Third, beyond examining the direct association between bullying victimization and Depressive Symptoms, this study further incorporated the mediating role of Internet Addiction and the moderating role of physical activity. This allowed for a more systematic examination of the underlying behavioral mechanisms through which bullying victimization may influence mental health outcomes among left-behind children. Compared with studies focusing on a single pathway, the proposed model provides a more nuanced explanation of the complex relationships among bullying victimization, Internet Addiction, physical activity, and Depressive Symptoms, thereby offering more targeted evidence for the development of comprehensive intervention strategies.

## Limitation

Several limitations should be acknowledged. First, physical activity levels were primarily assessed based on self-reports, which may be subject to recall bias and overestimation. Future studies should consider incorporating objective measurement tools, such as accelerometers, to more accurately assess the duration and intensity of physical activity. Second, the cross-sectional design of this study precludes causal inference among the variables. Longitudinal or intervention studies are needed to further validate the observed pathways. Third, although this study controlled for covariates such as Age and Gender, potential residual confounding may remain due to unmeasured factors, including family socioeconomic status, caregiver type, social support, and school environment. Future research should incorporate multi-source data and more rigorous study designs to minimize bias. Finally, Internet Addiction only partially mediated the association between bullying victimization and Depressive Symptoms, suggesting that other psychological or social mechanisms may also be involved. Future studies could further explore additional mediators, such as loneliness, self-esteem, emotion regulation, and social support, to more comprehensively elucidate the processes underlying mental health risks among left-behind children.

## Data Availability

The raw data supporting the conclusions of this article will be made available by the authors, without undue reservation.
